# Understanding Nursing Staff Perspectives on Fluid Balance Charting: A Nordic Focus Group Study

**DOI:** 10.1111/scs.70163

**Published:** 2025-12-07

**Authors:** Lisbeth Roesen Leinum, Marianne Krogsgaard, Sören Nordh, Jakob Lundager Forberg, Anders Ohlhues Baandrup, Nessn Azawi

**Affiliations:** ^1^ Department of Urology Zealand University Hospital Køge Denmark; ^2^ Faculty of Health and Medical Sciences University of Copenhagen Copenhagen Denmark; ^3^ Department of Surgery Zealand University Hospital Køge Denmark; ^4^ Department of People and Technology Roskilde University Roskilde Denmark; ^5^ Department of Emergency Medicine Helsingborg Hospital Helsingborg Sweden; ^6^ Department of Clinical Sciences Lund University Lund Sweden; ^7^ Department of Radiology Zealand University Hospital Køge Denmark; ^8^ Department of Urology Odense University Hospital Odense Denmark

**Keywords:** fluid balance chart, fluid intake‐output measures, fluid‐electrolyte balance, focus groups, hospital nursing staff, nursing records, physiologic monitoring, qualitative research, qualitative studies, water‐electrolyte balance

## Abstract

**Background:**

Evidence has established that the quality of fluid balance charting is often low, and quality improvement initiatives have had varying success. However, knowledge of the issue from a nursing staff perspective is lacking and has not previously been explored within a Nordic context. This study aimed to explore nursing staff's experiences with fluid balance charting in Denmark and Sweden.

**Methods:**

In this descriptive, qualitative study, data were collected through semi‐structured focus group interviews with nurses and healthcare assistants from various clinical departments in Denmark and Sweden. The interviews were analysed using a phenomenological‐hermeneutic approach inspired by Paul Ricoeur.

**Results:**

We included 25 nurses and healthcare assistants in eight focus group interviews. We found a notable discrepancy between the perceived importance and accuracy of fluid balance charting. Nursing staff perceived fluid balance charting as a fundamental nursing task and recognized that the quality of charting could directly impact patient outcomes. However, the involvement of multiple persons and high patient–nurse ratios caused nursing staff to experience the charting procedures as beyond their control and consider the results inaccurate. Quality may potentially be enhanced by following routines and responsibilities being clearly established. Digital technologies were suggested as a means of easing workflows.

**Conclusion:**

Although fluid balance charting is perceived as an essential nursing task potentially reducing mortality and morbidity, nursing staff lacked control over charting, which was frequently inaccurate. Enhanced charting procedures may be achieved through consensus on responsibilities, well‐established routines and the support of digital technologies.

## 1 Introduction

Maintaining fluid balance (FB) is critical to patients' health and recovery as disturbances lead to complications, prolong hospital stays and are associated with increased mortality [1]. Despite being a fundamental aspect of nursing care for decades, the reliability and adequacy of FB charting have been consistently questioned [2]. Although various quality improvement initiatives have been launched, the issue of inadequate FB charting remains a widespread challenge [3].

It is essential to understand nursing staff's perspectives on FB charting and the challenges they face in daily clinical practice. This insight will uncover the root causes of ongoing difficulties and pave the way for sustainable, long‐term improvements. These improvements are crucial for ensuring patients receive the highest standard of care possible. However, qualitative research exploring nursing staff's experiences of FB charting in a Nordic context has yet to be conducted. This research gap presents a significant opportunity to improve clinical practices and patient outcomes in the region.

A FB chart records an individual's intake and output over a 24‐h period, helping clinicians determine whether the patient is in a state of FB, deficit or overload [2]. While the concept may seem straightforward, the process is inherently complex due to several measures involved. Fluid input comprises oral fluids, intravenous medication and fluids, and enteral or parenteral nutrition, while outputs encompass urine, diarrhoea, stoma output, drainage, vomit, and nasogastric tube output [2].

Effective communication among staff members through accurate documentation of; for example, fluid intake and fluid losses, is a key indicator of quality of care as it allows for prompt treatment initiatives. By enabling treatment planning, FB charting, along with clinical examination and blood results, is crucial in determining a patient's hydration status across a wide range of medical and surgical specialties [4]. However, a recent systematic review has established that the proportion of completed FB charts was below 50% in more than half of the included studies. Despite numerous interventions aimed at improving charting practices, meaningful quality improvements remain rare [3].

Although FB charting is an established nursing responsibility, research has identified several barriers to its effectiveness. However, despite evidence of insufficient quality, only one qualitative study from the US [5] has examined nurses' experiences. We have otherwise limited insights into nursing staff's experiences and perceptions. Therefore, further exploration of nursing staff's attitudes and opinions is essential to develop sustainable solutions compatible with nursing practice and effectively address the challenges they face.

The healthcare systems in Scandinavia present unique characteristics, with public funding ensuring universal access. Healthcare delivery is increasingly shaped by technological advancements and a focus on digitalisation. Furthermore, the changing health needs of ageing populations, coupled with staff shortages, emphasize the urgent need for optimised healthcare practices. These characteristics highlight the need for research tailored to the specific context of Nordic healthcare.

In the Nordic context, where registered nurses (RNs) and healthcare assistants (HCAs) collaborate closely, FB charting is conducted in an interdisciplinary context. HCAs play a crucial role in FB charting in Denmark and Sweden, as they typically perform tasks related to eating and drinking. A narrow focus on RNs alone would overlook the contributions of HCAs, limiting the transferability of findings to clinical practice. Therefore, this study aimed to provide a comprehensive understanding of RNs' and HCAs' experiences with FB charting, examining the motivations, attitudes and behaviours shaping their practices.

The insights gained from this study will not only contribute to refining nursing practices but also have broader implications for healthcare policy and patient care management. By addressing the challenges nursing staff face in this critical area, we can enable more accurate FB charting, ultimately improving patient outcomes and enhancing the quality of care.

## 2 Aim and Objectives

This study aimed to explore nursing staff's perceptions of and experiences with FB charting and the significance of barriers and enablers related to FB charting.

The objectives were to:

1. Explore nursing staff's subjective experiences with FB charting.
2. Identify barriers and enablers in FB charting and their influence on charting quality as perceived by nursing staff.
3. Explore nursing staff's attitudes towards FB charting.

## 3 Methods

### 3.1 Design

We conducted a descriptive, explorative study using a qualitative approach to obtain an in‐depth understanding of nursing staff's experiences, perceptions and attitudes towards FB charting.

Data were collected through semi‐structured focus group interviews, which were recorded and transcribed. Seeking to avoid interpretation while interviewing, we applied a phenomenological‐hermeneutical methodology [6].

In reporting this study, we adhered to the Consolidated criteria for reporting qualitative research checklist (COREQ) [7].

### 3.2 Sampling and Recruitment

Head managers gave permission to recruit participants. Ward managers provided the names of potential participants, while others contacted us directly. Six participants were employed in the same department as the moderators. The inclusion criteria were RN or HCA with permanent in‐hospital employment and everyday experience in FB charting. No exclusion criteria were applied.

Interviewees were selected through purposive sampling [8] to ensure the representation of different departments and both RNs and HCAs in each focus group to balance the concepts of variation and homogeneity [9].

We ensured homogeneity by including participants who were employed in the same hospital, used the same electronic patient record, and shared experiences with FB charting. Including RNs and HCAs with long seniority and new graduates avoided creating overly homogenous groups [10]. Furthermore, to ensure variation and address different working flows and working conditions, nursing staff from surgical and medical wards, emergency departments and intensive care units (ICU) were included.

Focus group interviews were conducted with nursing staff from a Danish university hospital at two different sites, as well as from a public general hospital in Sweden, to ensure a Nordic perspective. All centres comprised a range of hospital specialties, including medical and surgical wards as well as ICUs. Although the healthcare systems in Denmark and Sweden are similar in many respects, differences exist. The electronic health record in the Danish hospitals automatically calculated fluid balance and was supplemented by mobile pocket devices, while in the Swedish hospital, fluid balance was entered manually into the electronic patient record.

### 3.3 Participants

We included 25 nursing staff in eight focus groups conducted in April and May 2023. Five RNs and five HCAs, respectively, dropped out due to high workloads, sickness, or other reasons. Four focus group interviews were conducted in each country, lasting on average 87 min (ranging from 77 to 95 min). Participants' median age was 38 years (range 23–60), and their professional seniority and employment in current departments varied from less than 1 year to more than 10 years. The majority were female (92%). Further characteristics of participants can be found in Table 1. The distribution of RNs and HCAs in focus groups is shown in Table 2.

**TABLE 1:** Characteristics of participants.

	*n* (%)
Sex	
Male	2 (8)
Female	23 (92)
Age (years) median, range	38 (23–60)
Education	
RN	17 (68)
HCA	8 (32)
Education (years)	
0–2	4 (16)
3–5	3 (12)
6–10	6 (24)
11–15	5 (20)
> 16	7 (28)
Employment in current department (yrs)	
< 1	9 (36)
2–4	11 (44)
5–10	3 (12)
> 11	2 (8)

Abbreviations: HCA, healthcare assistant; RN, registered nurse.

**TABLE 2:** Focus group participants.

Focus group	Country	RN/HCA	Alias
1	DK	1/1	A, B
2	DK	2/2	C, D, E, F
3	DK	2/1	G, H, I
4	DK	4/0	J, K, L, M
5	SWE	2/2	N, O, P, Q
6	SWE	2/1	R, S, T
7	SWE	1/1	U, V
8	SWE	3/0	W, X, Y

Abbreviations: HCA, healthcare assistant; RN, registered nurse.

### 3.4 Data Abstraction

Focus group interviews were moderated by the first (Danish female, PhD student) and third (Swedish male, MScN) authors, in their respective native languages having received training in qualitative interviewing and supervised by an experienced qualitative researcher. We collected data within our own institutions, and six participants were employed in the same department as the moderators. While our familiarity with the organisational structure deepened our understanding, it may have influenced participants' responses. We kept reflexive notes and held discussions among authors and observers to critically examine our assumptions.

To ensure all questions were discussed and make notes of non‐verbal communication, a further observer was present. The first author participated in all focus group interviews. All focus group interviews were audio‐recorded and transcribed verbatim. Swedish and Danish languages are mutually intelligible. However, to ensure full understanding of the Swedish transcripts they were thoroughly discussed with the third author, and parts of the transcripts were translated into Danish. Transcripts were not returned to participants. Sociodemographic variables were collected through a short questionnaire.

Focus group interviewing was chosen as this method is particularly suitable for exploring the range of opinions, perceptions and emotions concerning a specific topic. Participants formulate and clarify their views in interactional discussions [9], eliciting nuances in their perspectives [10, 11].

The recommended number of participants in a focus group varies [9, 11]. While large groups may challenge moderators and limit participants' opportunities to share experiences, smaller groups require sustained interaction to fulfil the requirements for focus group interviews [9]. Expecting last‐minute drop‐outs, we planned to over‐recruit by 20% [11] and prepared for pragmatic problem‐solving [9]. To ensure a convenient and undisturbed location, focus group interviews were conducted in a meeting room at the participants' place of employment. We strove to create an inclusive environment, encouraging participants to share their opinions and disagreements in a respectful tone, creating a comfortable room for communication to promote self‐disclosure [10]. To support interaction we further encouraged participants to comment on each other's statements [9, 10]. The interview guide consisted of three key questions developed based on the literature and discussions among researchers. An avenue of questioning was designed with questions introducing the topic and leading to the key questions. The questions were open‐ended, supported by interrelated, and logically coherent follow‐up questions [10].

Concluding the interview sessions, the moderator summarised the discussions to ensure that nothing was missed and that the points made were correctly understood [8, 10]. Following all focus group interviews, the first author made field notes regarding non‐verbal communication and participant interaction. The notes were used to reflect on the moderator's role and interactions between participants.

### 3.5 Data Analysis

According to Ricoeur, data analysis should seek to understand the meaning of a text rather than search for any hidden meaning behind the text or the author's intentions. Seeking to understand what the text (i.e., our transcripts) speaks about and follow the direction opened by it [12], our study intended to explore what the transcripts reveal about performing FB charting.

The data analysis process involved several stages in a circular process known as the hermeneutic circle. The analysis moved back and forth, from an initial understanding of the meaning as a whole through a structural analysis to a critical, in‐depth interpretation [12]. Divided into three methodological phases, a Ricoeur‐inspired analysis consists of a Naïve reading, a Structural analysis and a Critical interpretation leading to a comprehensive understanding [13].

The Naïve reading ensured an initial, spontaneous understanding of the meaning of the transcribed text as a whole [12]. Applying our intuition and with open minds, we approached the text to understand the phenomenon of FB charting as experienced by nursing staff [13].

The Structural analysis involved repeated readings of the transcripts to form an impression of “what is being said”. Having divided the text into units of meaning (quotations), these units were organised into 58 codes or subcategories, which were then structured into 14 categories and integrated into three themes that were narratively described [13]. “What the text speaks about” is explained in themes and compared to the naïve reading, which may be validated or rejected [13]. Our structural analysis was conducted in cooperation between the first and second authors using NVivo 14 (QSR International Pty Ltd., Australia).

In the critical interpretation, the themes were compared with relevant literature to reach the most suitable interpretation. By including other research, we deepened and expanded our understanding [13].

### 3.6 Ethical Considerations

The Regional Committee on Health Research Ethics deemed that no approval was required for this study (EMN‐2023‐02327). The Regional Data Protection Agency approved the study (REG‐010‐2023). Swedish regulations required no official approval. Having received written and oral information regarding the study aims and researchers' roles and titles, the participants provided written informed consent before the interview sessions.

## 4 Findings

Our initial understanding of the transcripts was based on a naïve reading (Table 3). In the structural analysis, we identified the following three themes: (1) FB charting is a fundamental nursing task relevant to target treatment, (2) FB charting is beyond individual control and inaccurate, and (3) Achieving consensus and simplifying charting methods is essential. Table 4 shows an example of the structural analysis while the three themes are presented below.

**TABLE 3:** Naïve reading.

Nursing staff regarded fluid balance charting as part of fundamental nursing care and an essential tool for clarifying patients' fluid status and guiding treatment decisions. The nursing staff had experienced the consequences of missed fluid balance charting on patient safety, and adequate fluid balance charting as a lifesaving medical tool. Nursing staff found that high patient–nurse ratios, the lack of routines and consensus among nursing staff and confusing and complex documentation challenged fluid balance charting. The challenges resulted in inadequate fluid balance charting, frustration and demanded mental strength on reconstructing data Nursing staff viewed fluid balance charting as a shared responsibility requiring clearly defined roles. Although nursing staff appreciated assistance from service staff, their involvement led to a lack of overview. Involving patients as co‐players was also perceived as a barrier to maintaining control over charting. It was nursing staff's experience that distinct communication among colleagues was required to secure efficient routines. Furthermore, the nursing staff considered digitalisation and technological aids to enhance future quality

**TABLE 4:** Structural analysis, an example.

A part of the structural analysis regarding the finding: Nursing staff consider fluid balance charting a fundamental nursing task relevant to target treatment		
**Unit of meaning**	**Unit of significance**	**Theme**
So, I think it's part of fundamental nursing to make sure that something goes in, and something goes out. That's insanely important, a significant part of fundamental care (A1) After all, [fluid balance charting] is part of fundamental nursing care (…). So, everyone should be aware of it (C2)	Fluid balance charting is a part of fundamental nursing and is used in evaluating patients' illness and treatment planning. Missed fluid balance charting may have serious consequences, but charting should always be combined with clinical assessment	Nursing staff consider fluid balance charting a fundamental nursing task relevant to target treatment
It [fluid balance charting] is a tool, just like the stethoscope is a tool, we need it to evaluate (…) It's very important (U7) You can say that you have control over the patient. If they have fluid balance charting in and out, you can see if there is an improvement or (…) nothing changes. We might have to change our plan as well. We may have to do something… (Q5) It's alpha and omega that we know what comes in and out, where we stand, and where things go wrong. Then, we can go back and see, well, it's because he drinks too much or we give him too many IV fluids, or he doesn't pee as he should (G3)		
It has the consequence, that they [patients] get pulmonary oedema or so… It physically affects the patient (K4) We have no idea [about their fluid balance], we just serve drinks all the time (…) Then we take [blood] samples of their kidneys, and it's disastrous. Maybe it was our fault because we [gave them intravenous fluids] and we didn't know they had been drinking (…) It can worsen their illness (N5)		
How strict do you have to be? There is no doubt that if you both weigh [patients] and record in‐ and output, look for oedema and look at the skin, then you could probably learn to be kinder to yourself [not judge yourself when you miss something] (A1) After all, you usually have a sense of your patients' fluid balance (M4) You can't just look at the chart, you have to look at the patient, the clinical picture of your patient (…) You have to turn your thoughts to ‘what do I have in front of me, and what does the paper show?’ The connection, I mean (S6)		

### 4.1 Theme 1: Fluid Balance Charting Is a Fundamental Nursing Task Relevant to Target Treatment

Nursing staff considered FB charting a fundamental nursing task that all nursing staff should know about as it is essential for the assessment of whether a patient is recovering or deteriorating. Nursing staff described FB charting as an indicator of the patient's well‐being and future course of illness: “It's a great indicator of where the patient is headed. If they eat and drink, they are usually healthier; if they don't eat and drink, they usually get sicker. It's a good predictor” (R6).

The relevance of FB charting and the necessary accuracy of its results depended on the patient category and severity of illness. Nursing staff maintained that in some patients, accurate hourly measurements are appropriate, while others only need their oral intake charted to ensure sufficient intake. The staff considered a clear indication for FB charting as essential. Further, doctors request and follow up on FB charting, which motivated nursing staff. However, they debated whether adherence to charting was improved by involving many rather than a few patients. Patients with heart failure, kidney diseases or sepsis, and those newly operated were considered high priority, and the significance of FB charting in critically ill patients was highlighted: “After all, we're doing it to save their lives” (G3). Charting was likewise required in patients with FB disturbances and abnormal fluid losses. Elderly and weak patients needed thorough attention.

Nursing staff considered that well‐performed FB charting clearly indicates patients' FB and thus enables targeted treatment: “It becomes very clear what the patient needs” (W8). FB charting was used to guide and adjust treatment continuously: “We constantly monitor and assess whether we should increase the administration [of intravenous fluids or medication]. We provide a status of the fluid balance … then we reassess if we should take action on that basis” (K4). Nursing staff stated that FB charting can help reveal the cause of a patient's disturbances, such as inappropriate administration of intravenous fluids or decreased urine output.

In contrast, poorly performed FB charting was deemed life‐threatening, capable of leading to unnecessary suffering and complications such as pulmonary oedema or deteriorating heart and kidney disease. Incomplete fluid charts were said to lead to possible mistreatment when treatment plans were based on inaccurate data. When nursing staff realized the consequences of missing FB charting, they acknowledged its importance and prioritized charting in the future. Some participants had not experienced the consequences of inadequate charting but were motivated by knowledgeable colleagues.

Nursing staff pointed to the link between FB charting and clinical assessment, stating that charting enables treatment adjustment before clinical signs appear, helping to prevent FB disturbance: “We're at the forefront through fluid balance charting of our surgical patients” (L4). Charting was not considered adequate by itself but should be accompanied by clinical evaluation and reflections on the agreement between charting data and the visible clinical signs. Nursing staff applied their clinical judgment in the evaluation and showed awareness that their assessment could be flawed.

### 4.2 Theme 2: Fluid Balance Charting Is Beyond Individual Control and Inaccurate

Aware that missing data could lead to unreliable FB charting, nursing staff questioned the trustworthiness of data: “Countless times, you've sat at night to summarise and had to write ‘incomplete list’ because so much data is lacking” (W8). Attempting to reconstruct data was experienced as cumbersome and like detective work. Others ascribed uncertain FB calculations to the missing time‐marking of documentation. Intravenous fluids were sometimes stopped prematurely without adjusting the given volume; likewise, oral fluids were frequently estimated rather than measured. Some nursing staff tried to catch up on charting at the end of shifts; however, relying on patients' recollections and estimations was (clearly) unreliable.

Increased nursing workloads were seen as having a decisive influence on charting quality: “How many patients each nurse is responsible for has a lot to do with how well they can focus on each patient and how much they do for them” (X8). Despite their intentions, nursing staff sometimes postponed fluid charting and ended up being unable to complete it. Following a shift where colleagues had failed to complete FB charting was frustrating and challenged mental capacity to regain control over patients' FB. Nursing staff deemed missing documentation unacceptable and unsafe for patients.

Nursing staff recognised the dilemmas of navigating conflicting demands and trusted their colleagues' intention to perform FB charting: “At first, I'd think, ‘Oh, that's really annoying’, but then again, they must've been too busy; something must have come up. (…) So, it's not that they're unwilling to get it done” (B1). However, some pointed out that not all colleagues considered keeping track of FB charts important enough. High workloads and the need to prioritise tasks affected the relationship between nursing staff and patients. Prioritising the sickest patients, nursing staff are forced to deprioritise others with the result that patients' needs are not always met.

The involvement of several staff members affected the oversight of patients' FB. While help from colleagues was welcomed, charting would be jeopardized if their colleagues were unaware of FB charting. In many wards service staff were employed to relieve nurses but lacked adequate healthcare training: “They don't know about [fluid balance] medically or diseases. I don't think they have the basic [knowledge]” (T6). When service staff failed to chart patients' intake, nursing staff lost control of FB.

Different paper charts for fluid intake, nutrition or pleural effusion were experienced as confusing and made it difficult to keep track. Where both a paper chart and an electronic patient record were used, overview and control suffered: “I think it's very simple. We shouldn't keep both a paper record and a digital record. It makes everything very complicated and difficult to follow” (N5).

Nursing staff preferred to retain control over patients' FB charting: “If the patients have no relatives, we can control their intake relatively well (…). What we can control, we do. And we do it for their own sake” (V7). Staff recognized the importance of patient involvement and invited patients as co‐players in their care, which was often enthusiastically welcomed. With responsibility for following up, nursing staff were nevertheless ready to intervene on FB if necessary. Only a minority of patients were able to perform FB charting sufficiently. This constituted an obstacle to maintaining control of charting.

### 4.3 Theme 3: Achieving Consensus and Simplifying Charting Methods Is Essential

Nursing staff pointed to a lack of established routines and consensus regarding the responsibility for FB charting: “We say it's everyone's job, so it easily becomes nobody's job. This could be part of the problem … that we don't dedicate the task to a specific person” (M4). Discussing their responsibility for informing HCAs about prescribed FB charting and the need for charting, RNs said they were held jointly responsible in case of inaccurate charting and viewed successful FB charting as dependent on collaboration between HCAs and RNs.

Several focus group participants described FB charting as completely unregulated and dependent on the individual staff member: “There's a lack of consensus in the group. If we discussed it, we would probably hear five or six different opinions on what should and should not be registered” (R6). In other departments, nursing staff promoted adherence to charting routines by sharing expectations among themselves: “There are mutual expectations that [fluid balance charting] should be done” (D2).

Establishing good routines and communication was seen as a way to enhance the quality of FB charting. Likewise, teaching new employees about the department's routines was considered helpful in maintaining consistency and consensus: “When someone starts working here, they follow some of us [with experience] to learn the routines. [How things are done] is often implicit and not put into words” (J4).

Nursing staff highlighted the benefits of a working environment where seeking and offering advice are part of clinical practice, paving the way for consensus and adherence to routines. Routines, such as bedside reports, daily weighing, serving fluid with every meal, nursing rounds, and calculating FB in every shift, were essential in maintaining charting quality and were expected to be complied with by all nursing staff. To ensure intravenous fluids were charted correctly, some nurses saved labels holding relevant information as a visual reminder until charting was completed. Others used posters, magnets or yellow paper charts as reminders.

However, consensus on routines and responsibilities was viewed as only partly resolving the issue of FB charting. Existing documentation methods were considered time‐consuming and bothersome, as obstacles to smoother workflows. Nursing staff thus stressed the need to reform the documentation process: “It feels like we need to find another type of system. (…) As I've mentioned before, digitisation! I think it would simplify things and make the whole process a lot easier” (V7).

Nursing staff were experienced in using technological tools for FB charting, for example, infusion pumps with automatic data transfer to the electronic patient record and mobile pocket devices enabling timely charting. However, complaints were voiced about the challenges of digital tools, such as time‐consuming login procedures and unnecessary steps. Some had experienced technical breakdowns: “I've been a real fan until the damn thing broke down” (H3). Despite everything, nursing staff were optimistic about technological aids and expected digital tools to expedite charting by simplifying the procedure. In the discussions, innovative ideas were offered to improve FB charting, such as using apps, intelligent toilets, speech recognition, etc.

## 5 Discussion

Exploring Nordic nursing staffs' experiences of FB charting, this study offers novel insights and identifies three main findings. Firstly, nursing staff consider FB charting an essential part of fundamental nursing care. Secondly, the involvement of numerous persons and time constraints challenges reliable FB charting. Thirdly, to enhance quality, nursing staff emphasise consensus regarding responsibilities and routines, as well as simplifying FB charting with novel technologies. This research not only emphasises the value of well‐known tools like education and established routines but also reveals a growing receptiveness among nursing staff towards technological solutions, signalling a transformative shift in their views and a recognition of the need for innovative solutions.

Nursing staff regarded FB charting as crucial to evaluate patients' hydration status and decide on treatment courses. A study involving 300 European nurses scored hydration issues highly important (6.7 on a 7‐point scale) [14]. Older studies have likewise pointed to the effectiveness of FB charting in managing FB and planning treatment [15, 16]. In contrast, an ICU study [17] found that, despite recognizing its value, only half of the nurses considered it as crucial as other patient care activities. These findings indicate that despite recognizing the significance of FB charting, nurses may deprioritize it in favour of other tasks; hence, continuous attention to FB charting is needed to maintain quality. Furthermore, concern has been voiced about unnecessary and prolonged charting without indication [18]. When a nursing task is perceived as redundant, it negatively impacts staff attitudes, underscoring a need for clear guidelines on when FB charting is warranted. To ensure proper prioritization of patients, we advocate applying guidelines and a systematic approach to identify patients in greater need of charting, thereby, avoiding redundant and excessive charting. See Table 5 for our practice recommendations. Further, extensive knowledge and advanced clinical skills among nursing staff must be ensured and staff resources better utilized to prevent either overlooking at‐risk patients or commencing redundant work [19]. Emphasizing the need for prioritization, our findings highlighted that the diagnosis and severity of illness should determine whether FB charting is lifesaving or aims to improve overall recovery.

**TABLE 5:** Recommendations for practice.

Maintain continuous attention to fluid balance charting from nursing staff Apply guidelines and a systematic approach to identify patients in need of charting Avoid unnecessary and prolonged charting without indication and make fluid balance charting in patients where indicated a top priority Provide equipment to enable fluid balance calculations and avoid imprecise estimations Adopt a person‐centred approach to target patients capable of participating in fluid charting Managers should support fluid balance charting by demonstrating ownership Provide continuous education and training for nursing staff and service staff Ensure consensus and constant alignment regarding routines Establish responsibilities by dedicating the task of fluid balance charting to specific staff members in daily practice Enhance communication and collaboration between colleagues and between nursing staff and service staff Ensure sufficient staff to uphold quality, retain nursing staff and prevent emotional exhaustion Implement technologies to take over routine tasks and ease the fluid balance charting

Nursing staff recognized that inaccurate FB charting involved a risk of mistreating patients [17], as shown in an audit where omitted fluid intake chartings led to excessive intake, a need for diuretics and uncertain decision‐making regarding intravenous fluid prescriptions [20]. Despite its acknowledged importance, nursing staff describe FB charting as unreliable due to missed chartings [20, 21]. Furthermore, inaccurate estimations cause flawed charting. Therefore, we recommend providing equipment that enables the accurate measurement and calculation of fluids. In the present study, maintaining control of FB charting proved challenging due to high workloads and demanding patient–nurse ratios. This aligns with previous findings [5, 17, 22] highlighting the patient–nurse ratio as the most significant barrier to accurate FB charting [5]. Nursing staff felt compelled to prioritize patients and tasks, causing them to feel unable to meet patients' needs. This corresponds to “implicit rationing of nursing care” as high workloads force nursing staff to ration nursing interventions, thus increasing the risk of adverse events and diminishing care quality. When nurses cannot meet patients' needs, it may lead to emotional exhaustion and impact job satisfaction [22]. We call for political action to ensure adequate staffing, uphold quality, retain nursing staff, and prevent emotional exhaustion.

The population of the Nordic countries is ageing, leading to increased healthcare needs. This demographic trend of a growing elderly population coincides with a reduced workforce, leading to future staff shortages [23]. Given these circumstances, it seems unlikely that the patient–nurse ratio will improve. Therefore, exploring alternative strategies, such as reallocating tasks and digitalisation, is imperative. Furthermore, most Nordic countries have implemented reforms aimed at increasing the healthcare sector's workforce [23].

Nursing staff observed that when several staff members are involved in serving fluids and charting, a lack of control may be the consequence. Research highlights that frequent caregiver changes [24] and inadequate communication between nursing and service staff hinder FB charting [25]. In Nordic healthcare, expanding the service staff's responsibilities to serve food and drinks was intended to relieve nursing staff. However, patient safety can be compromised when RNs are forced to delegate nursing tasks to untrained service staff. To address this, we recommend educating service staff, enhancing communication, and establishing routines; for example, using differently coloured trays for patients requiring FB charting.

Nursing staff were responsible for maintaining control of patients' FB and intervened when necessary. We learned that patients were invited to participate in FB charting, possibly motivated by hypotheses that involving patients would lead to more accurate charting. However, patients lacking motivation tended to forget or participate half‐heartedly, [20, 24]. Given that patients' participation influences charting accuracy [5], we recommend adopting a person‐centred approach to targeting patients capable of participating in FB charting.

Consensus regarding the routines and responsibilities of FB charting is critical to charting quality as a lack of ownership and accountability in FB charting causes non‐compliance with guidelines and inaccurate monitoring [19, 25]. While mandatory teaching of health care professionals with particular attention to the needs of service staff has been recommended [25], it is insufficient to achieve the desired goal. Sustainable quality improvement would require leaders to champion the change in word and action by demonstrating ownership. Likewise, involving key stakeholders' perspectives, effective communication, and continuous monitoring are called for to enhance quality [26]. Our participants also emphasised communication between colleagues and constant alignment of routines to maintain consensus. We suggest dedicating the task of FB charting to specific staff members and facilitating communication to ensure consensus.

Our study shows that nursing staff anticipate digital advancements and technological tools will enhance future charting quality by streamlining and simplifying workflows. Some had observed digital technologies improve patient safety, and they generally view technology positively despite common challenges in everyday nursing care, like time‐consuming logins, complicated systems and technological issues. Nurses likewise complained about time wasted through insufficient computer access or slow data loading [27]. A recent review elucidated issues affecting nurses' use of digital technologies and highlighted the impact of technologies on patient care as a driver for their willingness to adopt them [28]. Nurses insisted that unless systems are reliable, fast and accessible, they lead to frustration and stress, as also documented in this study.

The Nordic Region is at the forefront of digitalising the health sector and automation and technological adaptation are seen as key drivers to handle challenges [23]. Also nursing staff participating in this research expected technology to have a beneficial impact on FB charting. Hence, we advocate for implementing technologies to ease FB charting. However, to enable a digital transition, it is essential that technologies are compatible with clinical practices and that healthcare professionals gain the necessary digital skills.

### 5.1 Strength and Limitations

The qualitative approach offered insights into nursing staff experiences across clinical settings. Although variations in workflows, staffing, and working conditions might be considered a limitation, our findings elucidated similarities, supporting the transferability of our results to similar health systems. Focus group interviews yielded similar responses with minor nuances, indicating data saturation was achieved and the study objectives were met, although we acknowledge the possibility of something new emerging [29].

Both RNs and HCAs were included to capture diverse perspectives. While greater homogeneity within groups could have been achieved by forming focus groups of only RNs or HCAs, we considered groups composing both groups appropriate as they collaborate on FB charting in clinical practice. Unfortunately, not all focus groups included HCAs due to last‐minute cancellations. They are, however, well represented. To retain focus on charting fluids, we excluded other professional groups not actively involved in charting.

Recruitment challenges and last‐minute drop‐outs meant that two focus groups comprised only two participants; however, they can be described as focus groups yielding lively discussions and extensive interaction [9].

## 6 Conclusion

FB charting is a fundamental nursing task crucial for evaluating patients' FB and enabling the tailoring of fluid and pharmacological therapy. Helping to ensure targeted treatment, FB charting can potentially reduce mortality and morbidity, depending on the patient's diagnosis and illness severity. The focus group discussions, however, revealed that the accuracy of FB charting was often compromised by hasty estimations, the involvement of several people, high patient–nurse ratios and demanding workloads. Consensus on responsibilities and routines and integrating digital technologies may enhance charting by ensuring accuracy, timeliness and efficiency. Further, we recommend applying guidelines and a person‐centred approach to identify patients who need charting and are capable of participating in FB charting. Future research must explore evidence‐based strategies for elevating charting quality and developing intuitive digital technologies impacting nursing efficiency and patient outcomes. The influence of organisational structures and management on FB charting quality likewise needs further exploration.

## Author Contributions

All authors designed the study. The first and third authors conducted the interviews, and the first and second authors performed the analysis. All authors revised the manuscript.

## Funding

This research received funding from Interreg Öresund‐Kattegat‐Skagerrak (NYPSID 20358639).

## Ethics Statement

The Regional Committee on Health Research Ethics deemed that no approval was required for this study (EMN‐2023‐02327). The Regional Data Protection Agency approved the study (REG‐010‐2023). Swedish regulations required no official approval.

## Conflicts of Interest

The authors declare no conflicts of interest.

## Supporting information


**Appendix S1:** scs70163‐sup‐0001‐AppendixS1.docx.

## Data Availability

The data that support the findings of this study are available from the corresponding author, upon reasonable request. The data are not publicly available due to privacy or ethical restrictions.
