# Supplementary material for: Understanding Nursing Staff Perspectives on Fluid Balance Charting: A Nordic Focus Group Study
Source: Scand J Caring Sci. 2025 Dec 7;39(4):e70163. doi: 10.1111/scs.70163 (PMC12682938; doi:10.1111/scs.70163)
Supplement: Supplementary file 1 — Appendix S1: scs70163‐sup‐0001‐AppendixS1.docx. [file SCS-39-0-s001.docx]

**Interview guide**

| **Introduction**  Thank you very much for helping us learn more about fluid balance charting.  You all have one thing in common: you are employed at the hospital as a nurse or healthcare assistant and have experience in keeping fluid balance charts. At the same time, you are different and therefore also have different perspectives. We would hence really like to hear from you all.  Although there is a specific structure, we would like to have a free conversation where you can comment or build on something someone else has said.  It is also important to note that the purpose is not for us to agree; on the contrary, different points of view and perspectives are welcome.  Before we begin, I would like you to complete this brief questionnaire. We will use the information from the questionnaire to describe the group of participants. We would also like you to fill out a consent form. | | |
| --- | --- | --- |
|  | **Question** | **Follow-up question** |
| **Opening** | We will start with a round.  Could you please tell us your name, where you are employed, and briefly describe your role in fluid balance charting? |  |
| **Introduction** | How do you organise the fluid charting process in your department?  What does fluid balance charting consist of in your department? | Who is responsible?  How are tasks divided (between nurses/HCA/collaborators)?  Who initiates actions?  Is perspiration, weight, etc., included? |
| **Fluid balance charting in practice**  Barriers and enablers | What works well in relation to fluid balance charting?  What can promote high-quality documentation in fluid balance charting?  Is there anything that does not work optimally in fluid balance charting?  What barriers can hinder high-quality documentation in practice?  What significance do the conditions mentioned have for you as a staff member?  And for the patients? | What’s the easiest? And what makes it easy?  How does it influence quality?  What’s the most difficult? And what makes it difficult?  How does it influence quality?  Try to give an example?  Can you describe it?  Do conditions that make charting either easy or difficult affect your desire/motivation to commence fluid balance charting? |
| **Importance of fluid balance charting** | Do you think that keeping a fluid balance chart is useful?  How important is keeping a fluid balance chart compared to other nursing tasks?  Do you prioritize any particular patient groups?  Are all parts of the fluid balance chart equally important?  Is fluid record keeping a high priority in your department?  Is there an agreement among nursing staff about how high a priority fluid balance charting should be?  Are you influenced by (nursing) colleagues’ attitudes towards fluid balance charting?  Are you influenced by colleagues from other professional groups? How? | Why? / Why not?  E.g. Administrating medications, serving and documenting food and drinks, nutritional screening, personal hygiene, documenting wound car?  If not – what is most important? Least important?  How is that expressed? Who prioritizes it? Is there a difference in prioritization between professional groups? To whom is it most/least important?  How is that expressed?  In what way? Please give an example. E.g. documentation by colleagues? Documentation in all shifts?  For example, doctors’ focus on fluid balance charting during ward rounds? |
| **Improvements and dreams** | How can documentation of fluids balance be improved?  Do you think automating fluid balance charting would be possible? | If you should try to imagine the perfect method for monitoring fluid balance – what would it look like? |
| **Closing** | Summary:  We have talked about fluid balance charting as…  It is generally organized…  It works well to…  Barriers are…  This means that…  Fluid balance charting is prioritized….  Is this an appropriate summary?  Is there anything we are missing? | Others say the electronic patient record/calculations errors/equipment can influence charting? What do you think about that? |
| **Closing remarks and thank you for today**  Thank you very much for participating today.  It was exciting to hear about your different experiences and perceptions of keeping fluid balance charts. It was very useful!  If any of you are interested in receiving the paper we will write based on, among other things, our conversations today, please let us know. | | |
